# Jasmonate-Elicited Stress Induces Metabolic Change in the Leaves of *Leucaena leucocephala*

**DOI:** 10.3390/molecules23020188

**Published:** 2018-01-24

**Authors:** Yingchao Xu, Zhenru Tao, Yu Jin, Shuangyan Chen, Zhongyu Zhou, Amy G. W. Gong, Yunfei Yuan, Tina T. X. Dong, Karl W. K. Tsim

**Affiliations:** 1 Key Laboratory of Plant Resources Conservation and Sustainable Utilization, Guangdong Provincial Key Laboratory of Applied Botany, South China Botanical Garden, Chinese Academy of Sciences, Guangzhou 510650, China; yingchaoxu@scbg.ac.cn (Y.X.); taozhenru@outlook.com (Z.T.); jinyu@scbg.ac.cn (Y.J.); shuangyanchen@scbg.ac.cn (S.C.); yuanyunfei@scbg.ac.cn (Y.Y.); 2 University of Chinese Academy of Sciences, Beijing 100049, China; 3 College of Light Industry and Food Sciences, Zhongkai University of Agriculture and Engineering, Guangzhou 510225, China; 4 Division of Life Science and Center for Chinese Medicine, The Hong Kong University of Science and Technology, Hong Kong, China; amygong90@gmail.com (A.G.W.G.); botina@ust.hk (T.T.X.D.); botsim@ust.hk (K.W.K.T.)

**Keywords:** *Leucaena leucocephala*, NMR, principal component analysis, partial least squares discriminant analysis, elicitors, environmental stress

## Abstract

The plant *Leucaena leucocephala* was exposed to four jasmonate elicitors, i.e., jasmonic acid (JA), methyl jasmonic acid (MeJA), jasmonoyl-l-isoleucine (JA-Ile) and 6-ethyl indanoyl glycine conjugate (2-[(6-ethyl-1-oxo-indane-4-carbonyl)-amino]-acetic acid methyl ester) (CGM). The treatment was to mimic the herbivores and wounding stresses. By using NMR spectroscopy along with chemometric analysis, including principal component analysis (PCA) and partial least squares discriminant analysis (PLS-DA), the changes of metabolites in the leaves of *L. leucocephala* were determined under the stress as induced by the four elicitors. The challenge of JA-Ile caused an accumulation of lactic acid (**6**), *β*-glucose (**10**), alanine (**12**), threonine (**13**), steroids (**18**), 3,4-dihydroxypyridine (**19**) and an unidentified compound **20**. The chemometric analysis of the PCA and PLS-DA models indicated that the alternation of metabolites triggered by JA, MeJA, and CGM treatments were very minimum. In contrast, the treatment by JA-Ile could induce the most significant metabolic changes in the leaves. Moreover, there was very minimal new metabolite being detected in responding to the jasmonate-induced stresses. The results showed some metabolite concentrations changed after application of the elicitors, which may be related to a high level of tolerance to stress conditions as well as the strong ecological suitability of *L. leucocephala*.

## 1. Introduction

*Leucaena leucocephala* belongs to the family Fabaceae: the leaves and pods of this plant are a favorite forage crop for cattle and sheep. *L. leucocephala* plant was widely distributed throughout tropic and subtropic areas. Due to its extraordinary ecological suitability and ability to grow quickly, this plant has been widely cultivated for ecological restoration construction and expressway slope improvement in China. The outstanding ecological suitability of *L. leucocephala* indicated strong resistance against the environmental stresses, including drought, herbivores, and wounding. Six stress-related genes and 22 hypothetical proteins in *L. leucocephala* were identified and expressed at high levels under drought conditions [1].

This has been well-established that endogenous jasmonic aicd (JA), methyl jasmonate (MeJA) and jasmonoyl-l-isoleucine (JA-Ile), play a prominent role in promoting plant defense in responding to environmental stresses, e.g., herbivores and wounding [2,3,4]. Coronalon, a synthetic 6-ethyl indanoyl isoleucine conjugate, was reported to be a highly active stress inducer mimicking jasmonate being involved in insect and disease resistance [5,6]. 2-[(6-Ethyl-1-oxoindane-4-carbonyl) amino] acetic acid methyl ester (CGM), a new analogue of coronalon, was designed and synthesized by Tan [7]. The isoleucine residue in coronalon was replaced by glycine in CGM for a better water solubility. The plant hormone jasmonates are a class of oxylipin compounds [8]. Exogenous application of these jasmonates on plant was able to simulate herbivory and wounding stresses [9]. For example, the total phenolic content, including rosmarinic acid, caffeic acid, eugenol and linalool, was markedly increased after the treatment of MeJA on sweet basil (*Ocimum basilicum*) plant [10]. The treatment of MeJA increased the accumulation of terpenoid indole alkaloids in *Catharanthus* and *Cinchona* plants [11,12], and enhanced the production of secondary metabolites in tomato tissue [13]. Coronalon and MeJA promoted the biosynthesis of lignans in suspension cultures of *Linum nodiflorum* [6]. On the other hand, CGM triggered volatile biosynthesis in the lima bean and ginkgo leaves [7].

**Figure 1:**
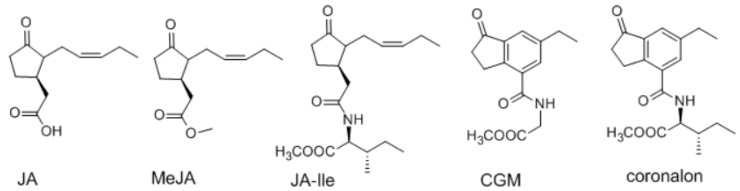
The structures of four jasmonate elicitors used in this research and coronalon (not used here).

In the scenario of *L. leucocephala*, the amount of mimosine was increased locally by application of mechanical damage (simulating herbivory) in shoots [14]. Salicylic acid (simulating pathogen attack) applied in culture media of *L. leucocephala* induced mimosine accumulation in roots [14]. The *L. leucocephala* plant was well-known for its high level of tolerance to various stress conditions [1]. However, the study using jasmonate-elicited stress in *L. leucocephala* was very limited. To reveal the possible metabolic alternation under stress condition, four exogenous jasmonates, including JA, MeJA, JA-Ile and CGM (Figure 1) were being used as elicitors, and were sprayed on *L. leucocephala* to induce a condition of herbivores and wounding stresses. After the exposure, the change of metabolites in *L. leucocephala* leaves were examined by NMR spectroscopy along with chemometric analysis, including principal component analysis (PCA) and partial least squares discriminant analysis (PLS-DA).

## 2. Results and Discussion

Nine MeJA-treated plant samples, nine JA-treated samples, eleven JA-Ile-treated samples, twelve CGM-treated samples and eleven control (labeled as CK) samples were collected for analyses. The aqueous CD_3_OD extract of total 52 *L*. *leucocephala* samples were subjected to NMR analyses. Seven organic acids, malic acid (**1**), citric acid (**2**), formic acid (**3**), succinic acid (**4**), RC(OH)CH_3_-COOH (**5**), lactic acid (**6**) and fumaric acid (**7**), four carbohydrates, sucrose (**8**), *α*-glucose (**9**), *β*-glucose (**10**), and fructose (**11**), three amino acids, alanine (**12**), threonine (**13**), and mimosine (**14**), two flavonoids, quercetin (**15**) and quercetin-3-*O*-*α*-rhamnoside (**16**), and three miscellaneous compounds, choline (**17**), steroids (**18**), and 3,4-dihydroxypyridine (**19**) were identified by 1D and 2D NMR spectra and comparison with the Biological Magnetic Resonance Data Bank (BMRB) database [15]. The structures of those 19 identified metabolites were shown in Figure S2 (Supplementary Material). Moreover, the areas of two obvious single peaks (*δ*_H_ 2.09 and 2.72) referred to two unidentified compounds (**20** and **21**) were integrated and included in the chemometric analysis. Diagnostic peaks of compounds **1**–**21** were shown in Table 1.

**Figure 2:**
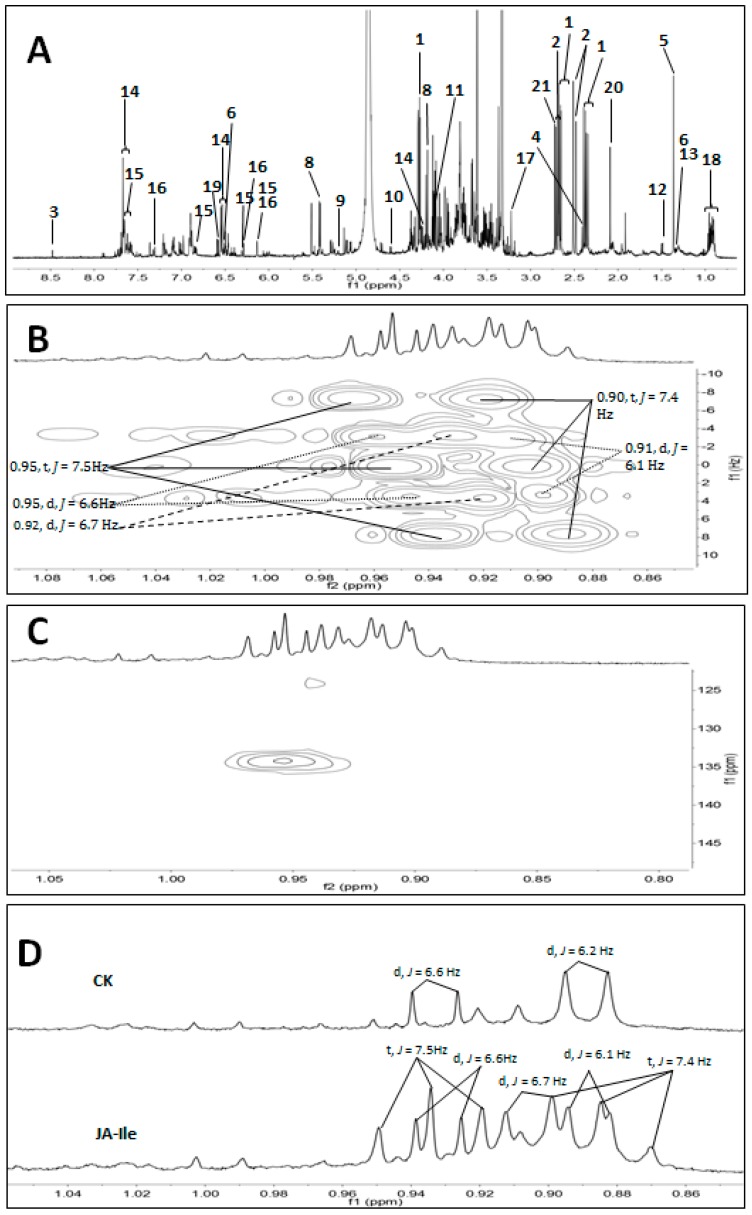
1D and 2D NMR spectra of *L. leucocephala* leaf extracts. (**A**) Representative ^1^H-NMR spectrum of *L. leucocephala* leaf extracts. Peaks: **1**, malic acid; **2**, citric acid; **3**, formic acid; **4**, succinic acid; **5**, RC(OH)CH_3_-COOH; **6**, lactic acid; **7**, fumaric acid; **8**, sucrose; **9**, *α*-glucose; **10**, *β*-glucose; **11**, fructose; **12**, alanine; **13**, threonine; **14**, mimosine; **15**, quercetin; **16**, quercetin-3-*O*-*α*-rhamnoside; **17**, choline; **18**, steroids; **19**, 3,4-dihydroxypyridine; **20**, unidentified; **21**, unidentified; (**B**) *J* values were determined in the 2-dimensional *J*-resolved NMR spectroscopy of JA-Ile treated plants; (**C**) HMBC correlation between methyl signals and olefinic carbon at *δ*c 134.2; (**D**) A set of methyl signals at 0.85-1.05 ppm in JA-Ile elicited *L. leucocephala* leaf extracts was compared to control (CK).

Representative ^1^H-NMR spectrum of *L. leucocephala* leaf extract was shown in Figure 2A. The ^1^H-NMR spectrum exhibited signals at *δ*_H_ 2.67 (dd, *J* = 15.3, 3.0 Hz, 1H), 2.37 (dd, *J* = 15.3, 10.0 Hz, 1H), and 4.28 (dd, *J* = 10.0, 3.0 Hz, 1H), which were diagnostic for malic acid (**1**). The ^1^H-NMR spectrum showed two double peaks at *δ*_H_ 2.50 (d, *J* = 15.6 Hz) and 2.69 (d, *J* = 15.6 Hz), combined with HMBC correlation between these proton doublet with carbons at *δ*c 45.7, 75.1, 179.0, 182.0, and citric acid (**2**) was consequently determined. Formic acid, succinic acid and fumaric acid were identified on the basis of proton resonance at *δ*_H_ 8.46 (1H, s), 2.41 (s) and 6.50 (1H, s), respectively. A tertiary methyl at *δ*_H_ 1.36 showed HMBC correlations with an oxygenated carbon (*δ*c 77.0) and a carboxyl (*δ*c 180.9), which implied the partial structure of **5**. Lactic acid was determined by the observation of a double peak at *δ*_H_ 1.34 (d, *J* = 6.8 Hz).

The proton resonances at *δ*_H_ 5.40 (d, *J* = 3.8 Hz, 1H) and 4.17 (d, *J* = 8.7 Hz, 1H) were diagnostic for anomeric proton of *α*-glucose and CH-3 of fructose in sucrose, respectively. *α*-Glucose was confirmed on the basis of anomeric proton doublet at *δ*_H_ 5.21 with a small ^3^*J*_H-1,H-2_ coupling constant (3.8 Hz) and Heteronuclear Single Quantum Coherence (HSQC) correlation between *δ*_H_ 5.21 and *δ*c 92.6. In the same way, *β*-glucose was identified based on the observation of anomeric proton doublet at *δ*_H_ 4.60 with a large ^3^*J*_H-1,H-2_ coupling constant (7.9 Hz), combined with HSQC correlation between *δ*_H_ 4.60 and *δ*c 96.2. Fructose was detected on the basis of CH-3 at *δ*_H_ 4.09 (d, *J* = 3.5 Hz, 1H) and corresponding HSQC correlation with *δ*_C_ 77.6. The proton signal at 1.49 ppm (d, *J* = 7.3 Hz) showed HMBC correlation with *δ*c 175.5 and 50.5; alanine was therefore elucidated. Threonine was identified on the basis of methyl proton signals at *δ*_H_ 1.32 (d, *J* = 6.6 Hz, 1H). Choline was elucidated on the basis of three exactly identical methyl signals at *δ*_H_ 3.22 (9H, s). ^1^H-NMR signals at 7.69 (overlapped), and 6.60 (d, *J* = 7.2 Hz, 1H), were owed to 3,4-dihydroxypyridine (**19**), which was a degradative derivative of mimosine (**14**). A set of methyl signals of double peaks (0.95, *J* = 6.6 Hz; 0.92, *J* = 6.7 Hz; 0.91, *J* = 6.1 Hz) and triple peaks (0.95, *J* = 7.5 Hz; 0.90, *J* = 7.4 Hz) were shown in JA-Ile-treated plants, and of which *J* (coupling constant) values were clearly determined in the 2-dimensional *J*-resolved NMR spectroscopy (Figure 2B). One of above methyl groups showed HMBC correlation with olefinic carbon at *δ*c 134.2 (Figure 2C), which was appeared in JA-Ile-treated plant and absent in the HMBC spectra of JA-, MeJA-, CGM-treated and control plants. Figure 2D showed that the methyl at 0.95 (t, *J* = 7.5 Hz), 0.90 (t, *J* = 7.4 Hz), 0.92 (d, *J* = 6.7 Hz) were newly appeared signals in JA-Ile-treated plant. These methyl signals might be contributed to steroids (**18**), and carbon at *δ*c 134.2 should be ascribed to the C-22 of steroids. 

Mimosine (**14**), quercetin (**15**) and quercetin-3-*O*-*α*-rhamnoside (**16**), were the main organic component with small molecular weight in *L. leucocephala* leaf, which were obtained by our extraction and isolation experiments. The ^1^H and ^13^C-NMR spectra of mimosine (**14**), quercetin (**15**) and quercetin-3-*O*-*α*-rhamnoside (**16**) were shown in Figure S3 (Supplementary Material). Since the ^1^H-NMR spectra of leaf extracts were carefully compared with the authentic spectra at hand in Figure S3, and thereby mimosine (**14**) [16], quercetin **(15**) [17] and quercetin-3-*O*-*α*-rhamnoside (**16**) [18] could be unambiguously determined in the elicited *L*. *leucocephala* leaves. In detail, the signals at *δ*_H_ 7.65 (overlapped), 6.54 (d, *J* = 6.7 Hz, 1H), 4.34 (dd, *J* = 14.2, 5.0 Hz, 1H), 4.23 (dd, *J* = 14.2, 6.7 Hz, 1H) were ascribed to mimosine (**14**). ^1^H-NMR signals at *δ*_H_ 7.51 (d, *J* = 2.1 Hz, 1H), 7.47 (dd, *J* = 8.6, 2.1 Hz, 1H), 6.89 (d, *J* = 8.5 Hz, 1H), 6.31 (d, *J* = 1.8 Hz, 1H), and 6.13 (d, *J* = 1.8 Hz, 1H) were referred to quercetin (**15**). ^1^H-NMR signals at *δ*_H_ 7.36 (d, *J* = 2.1 Hz, 1H), 7.32 (dd, *J* = 8.4, 2.1 Hz, 1H), 6.91(d, *J* = 8.4 Hz, 1H), 6.29 (d, *J* = 1.9 Hz, 1H), 6.13 (d, *J* = 1.9 Hz, 1H), and 0.94 (d, *J* = 6.2 Hz, 3H) were contributed to quercetin-3-*O*-*α*-rhamnoside (**16**). 

**Figure 3:**
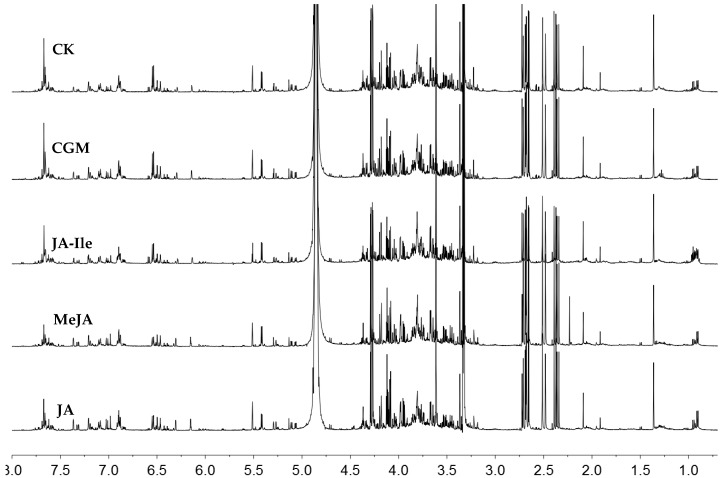
^1^H-NMR spectra comparison of *L. leucocephala* leaf extracts under different elicitors.

The metabolic profiles of elicited *L. leucocephala* leaves, i.e., the treatments of CGM, JA-Ile, MeJA and JA, were not drastically altered but certain metabolites have differential abundances. Metabolites in the ^1^H-NMR spectra comparison of *L. leucocephala* leaf extracts under the four stress elicitors were shown to be at different intensity (Figure 3). The relative contents of nineteen determined and two unidentified compounds in each treatment were shown in Table S1 (Supplementary Material), and which was imported into SIMCA-P software for chemometric analyses. Principle component analysis (PCA) was the most basic and efficient method to analyze complex data in metabolomics, which could extract and display systematic variations from the data, as well as to detect the grouping, trend and outlier [19]. Each point in a PCA score plot represented a single sample, and the sample clustered together was considered to have similar characteristic, i.e., similar metabolic profiling. For assessing the potential variables correlating to metabolite contents, PCA was applied aiming to observe cluster of *L. leucocephala* leaf extracts under different elicitation.

^1^H-NMR spectra comparison of leaves extracts of *L. leucocephala*, which were treated by MeJA (0.5 mM), JA (0.5 mM), JA-Ile (0.5 mM), CGM (0.5 mM) and control (labeled as CK), respectively. The metabolite profile of elicited *L. leucocephala* leaves were not drastically altered but certain metabolites have differential abundances. 

**Figure 4:**
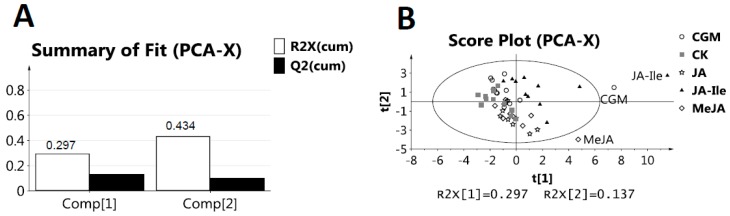
Principal component analysis (PCA) of metabolites in *L*. *leucocephala* leaf extracts under different elicitations. (**A**) The summary of fit of PCA score plot with two components was calculated. The first and second components accounted for 43.4 % (29.7 and 13.7 %, respectively) of the overall variance; (**B**) PCA score plot discriminated *L. leucocephala* extracts from JA-Ile (triangle) and control treatment (CK, box), and was disabled to distinguish between JA (star), between MeJA (diamond), between CGM (circle) and control treatment (box), respectively. There are three outliers, one from MeJA, one from JA-Ile and another one from CGM.

**Figure 5:**
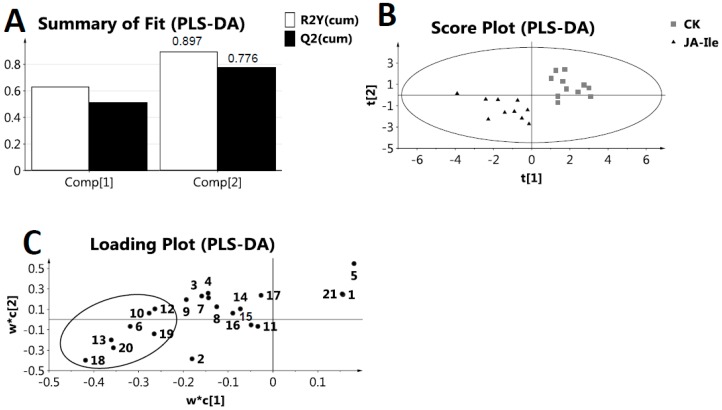
Partial least squares discriminant analysis (PLS-DA) of metabolites in *L*. *leucocephala* leaf extracts under JA-Ile elicitation. (**A**) The summary of fit of PLS-DA model of JA-Ile and control. The cumulative R2Y and Q2 were 0.897 and 0.776 respectively, when two components were calculated; (**B**) PLS-DA score plot discriminated *L. leucocephala* extracts from JA-Ile (triangle) and control treatment (CK, box) more clearly than PCA score plot; (**C**) PLS-DA loading plot. The notations of 1-21 were corresponding to different metabolites as listed in Table 1. JA-Ile caused an accumulation of lactic acid (**6**), *β*-glucose (**10**), alanine (**12**), threonine (**13**), steroids (**18**), 3,4-dihydroxypyridine (**19**) and an unidentified compound **20**.

The cumulative R2X for the first and the second components were 0.297 and 0.434, respectively (Figure 4A), which indicated that the first and the second components of PCA score plot accounted for 43.4 % (29.7 and 13.7 %, respectively) of the overall variance. Figure 4B showed three outliers, and exhibited an unclear cluster between the plant treatments of JA, between MeJA, between CGM and control samples, respectively. In contrast, a clear cluster between JA-Ile and control treatments was identified. To further determine the metabolite variable playing an important role in discriminating *L*. *leucocephala* leaf extracts from each elicited and control samples, PLS-DA was therefore performed. The cumulative R2Y and Q2 were 0.897 and 0.776, respectively, in the PLS-DA model of JA-Ile and control (CK) (Figure 5A). In parallel, the cumulative R2Y and Q2 were 0.824 and 0.587, respectively for JA, 0.757 and 0.242 for MeJA, and 0.619 and 0.385 for CGM (Figure S4, Supplementary Material). Having 200 permutations and two components, the permutations plot was performed in order to validate PLS-DA model. Figure S5 (Supplementary Material) revealed positive slopes and minus Q2 values of y-intercept for four elicitors. However, it was not found that all R2 and Q2 values to the left were lower than the original points to the right for JA, MeJA and CGM treatments, which indicated that the PLS-DA model was not a stably valid model, respectively. Nevertheless, it was clear that all R2 and Q2 values to the left are lower than the original points to the right for JA-Ile treatment, which indicated that the PLS-DA model was a valid model. The aforementioned information suggested that the JA-Ile treatment showed the most significant metabolic changes among the four elicitors. It was reported that MeJA and JA might be activated by having a conjugation to isoleucine, and thus JA-Ile was the potential active form in triggering the function [20,21]. It was also reported that the amplitude and duration of JA responses were regulated largely by the intracellular level of JA-Ile [22]. Therefore, our finding was consistent with previous reports [20,21,22]. The insensitive of JA, MeJA, and CGM treatments in inducing metabolic changes could be by several possible reasons: (i) *L. leucocephala* plant was different from other plants such that they are not able to have a conjugation with isoleucine; and (ii) the treatment time was too short and not enough time for the isoleucine conjugation.

As shown by the PLS-DA score plot (Figure 5B), the JA-Ile-treated samples were clearly separated from the control sample. From the loading plot (Figure 5C), it was found that JA-Ile could cause an increase of lactic acid (**6**), *β*-glucose (**10**), alanine (**12**), threonine (**13**), steroids (**18**), 3,4-dihydroxypyridine (**19**) and an unidentified compound **20**. Lactic acid (**6**) is the product under anaerobic respiration in plants, which may be related with extra energy consumed under environmental stress, as induced by JA-Ile. Amongst these metabolites, steroids (**18**) are important components of cell membranes, which could alter membrane fluidity and protein environment [23]. Steroids could have a high production rate under JA-Ile treatment, therefore steroids might affect membrane functions of the plant in order to cope with environmental stresses.

It was reported previously that the amount of mimosine was increased locally by mechanical damage (simulating herbivory) applied to shoots of *L. leucocephala* [14]. Salicylic acid (simulating pathogen attack) applied in culture media of *L. leucocephala* stimulated mimosine accumulation in roots [14]. 3,4-Dihydroxypyridine (**19**) was accumulated under JA-Ile elicitation. Contrasting to accumulation of mimosine under salicylic acid elicitation, JA-Ile and salicylic acid might activate different defense pathways in *L. leucocephala*, as to resist outside stress. 3,4-Dihydroxypyridine is the degradative product of mimosine [24], which is the major components in *L*. *leucocephala*. Mimosine was considered as an allelochemical and a potent bio-herbicide [25], which was supposed to be induced under elicitation. 3,4-Dihydroxypyridine is also a toxic molecule of *L*. *leucocephala* forage toxicosis, which is a potent goitrogen in ruminants [26,27]. Plant secondary metabolites were produced constitutively or were inducible [28]. Mimosine belongs to constitutive metabolite, while 3,4-dihydroxypyridine belongs to inducible metabolite, which therefore could be induced and accumulated under JA-Ile treatment. Moreover, JA-Ile elicitation might activate the degradative pathway of mimosine into 3,4-dihydroxypyridine. 

The rest of increased metabolites, including *β*-glucose, alanine and threonine are common primary metabolites in plants; however, the causes of such increase under JA-Ile treatments have not been resolved. In summary, the profiles of metabolites of the elicited *L*. *leucocephala* leaves had not been drastically altered. There was very minimal new metabolite being detected in responding to the jasmonate-induced stresses, as simulated by the four jasmonates elicitors. It was just found that the contents of some metabolites appeared to have differential abundances, which might be related to high level of tolerance to stress condition and strong ecological suitability of *L. leucocephala*.

## 3. Materials and Methods 

### 3.1. Chemicals and Reagents 

D_2_O containing TSP (0.05% wt), and CD_3_OD, MeJA and (±) JA were purchased from Sigma-Aldrich (St. Louis, MO, USA). Na_2_HPO_4_, NaH_2_PO_4_·H_2_O were purchased from Zhiyuan Biological Technology (Tianjin, China). JA-Ile was prepared from (±) JA and isoleucine according to a previously established method [29]. CGM was provided by Prof. J.W. Tan [7].

### 3.2. Plant Growth, Treatment and Harvest 

The *L. leucocephala* seeds were bought from Tian Ye Feng Landscaping Co., Ltd. (Guangzhou, China). The seeds were immersed into boiling water, and broke dormancy. Disposed seeds were sprouted at plate medium with wet filter paper. Sprouted *L*. *leucocephala* were transferred into pots with nutrient soil and grown for about 4 weeks (16 h light/8 h dark cycle). The growing *L. leucocephala* plants were shown in Figure S1 (Supplementary Material). The *L. leucocephala* plants having the fifth to sixth true leaves were sprayed with the four stress elicitors (JA, MeJA, JA-Ile and CGM), each at 0.5 mM concentration in water containing 0.1% CH_3_OH for better solubility. Spraying was applied at two continuous days, three times a day. The seedlings for MeJA treatment were taken out from the growth room, sprayed in an open area, covered with a transparent plastic lid before moving back to the growth room. Control plants were treated with water containing 0.1% CH_3_OH. Leaves were harvested at one day after elicitor’ treatment (*n* = 9 for JA and MeJA treatments, respectively; *n* = 11 for JA-Ile and control treatments, respectively; *n* = 12 for CGM treatment). The harvested aerial parts of plants were immediately frozen in liquid nitrogen, of which the leaves were homogenized under liquid nitrogen and lyophilized to dry powders.

### 3.3. Sample Preparation

D_2_O (pH = 7.4) buffer was prepared according to literature [30] with minor adjustment, which in brief was that D_2_O was fully used instead of adding some H_2_O, containing 28.85 mg/mL Na_2_HPO_4_, 6.825 mg/mL NaH_2_PO_4_·H_2_O, and 1 mM TSP. Thirty mg powder of *L*. *leucocephala* leaves was exactly weighted, in which 0.5 mL CD_3_OD and 0.5 mL D_2_O (pH = 7.4) containing TSP (0.05% wt) as internal chemical shift and content standard, were added. After that, the samples were sonicated for 30 min and centrifuged at 14,000 rpm for 5 min. The supernatant was transferred to a standard 5-mm NMR tube for NMR experiments.

### 3.4. NMR Experiments

The ^1^H-NMR spectra were recorded on a BRUKER AVANCE III 500 spectrometer (Bruker, Karlsruhe, Germany) at 500.13 MHz proton frequency, equipped with Z-gradient system at 25 °C. All the NMR data was obtained under an automatic procedure, which was performed by collecting 128 scans of 32,768 data points for each spectrum. The spectral width was 7500 Hz, and the relaxation delay was set to 1.0 sec with a 4.4 sec acquisition time. Consequently, each sample required about 11 min. The chemical shifts of all the spectra were calibrated using the signals from TSP-d_4_ in D_2_O at *δ* 0.00, respectively.

For the purpose of signal assignment, standard 2 dimensional NMR spectra were acquired at 25 °C, including ^1^H-^13^C heteronuclear single quantum coherence spectroscopy (HSQC), heteronuclear multiple bond correlation (HMBC) and J-resolved spectroscopy (JRES). ^1^H-^13^C HSQC and HMBC experiments were recorded by using the gradient selected sequences with 512 transients and 2048 data points for each of the 128 increments. The spectral widths were set at 6,000 Hz for 1H, 20,625 Hz for ^13^C in HSQC experiments and 27,500 Hz for ^13^C HMBC experiments, respectively. For JRES spectra, 128 transients were collected into 4096 data points for each of the 80 increments with a spectral width of 6000 Hz in the acquisition and 60 Hz in the evolution dimensions. The data were zero-filled to a 2000 × 2000 matrix with appropriate window functions prior to Fourier transformation.

### 3.5. NMR Data Processing and Multivariate Data Analysis

The ^1^H-NMR spectra of *L. leucocephala* leaf extracts were manually corrected for phase and baseline distortions by using software package TOPSPIN (v3.2, Bruker, Billerica, Massachusetts, USA). Nineteen metabolites were identified by 1D and 2D NMR spectra. The area of characteristic peak for each metabolite was integrated. The relative contents of these nineteen determined and two unidentified compounds were defined by comparing the characteristic resonance integral of each molecule with the TSP signal normalized for nine protons generating the resonance. A linear baseline scaling normalization approach was used. The baseline was constructed by calculating the median of each feature over all spectra. The scaling factor was computed for each spectrum as the ratio of mean intensity of baseline to mean intensity of spectrum. The intensities of all spectra were multiplied by their particular scaling factors [31]. Multivariate statistical analyses, including principal component (PCA) and orthogonal partial least squares discriminant analysis (PLS-DA), were performed using SIMCA-P version 13.0 software (Umetrics, Umeå, Sweden) having mean centering and unit variance scaling. The relative contents of nineteen determined and two unidentified compounds in each treatment were shown in Table S1, which was imported into SIMCA-P software for chemometric analyses.

## 4. Conclusions

The plant *Leucaena leucocephala* was exposed to four jasmonate elicitors, i.e., jasmonic acid (JA), methyl jasmonic acid (MeJA), jasmonoyl-l-isoleucine (JA-Ile) and 6-ethyl indanoyl glycine conjugate (2-[(6-ethyl-1-oxo-indane-4-carbonyl)-amino]-acetic acid methyl ester) (CGM). The treatment was to mimic the herbivores and wounding stresses. The challenge of JA-Ile caused an accumulation of lactic acid (**6**), *β*-glucose (**10**), alanine (**12**), threonine (**13**), steroids (**18**), 3,4-dihydroxypyridine (**19**) and an unidentified compound **20**. The chemometric analysis of the PCA and PLS-DA models indicated that the alternation of metabolites triggered by JA, MeJA, and CGM treatments were at minimum. In contrast, the treatment by JA-Ile could induce the most significant metabolic changes in the leaves. Moreover, there was very minimal new metabolite being detected in responding to the jasmonate-induced stresses. The results showed the contents of some metabolites appeared to be change, which may be related to high level of tolerance to stress conditions as well as the strong ecological suitability of *L. leucocephala*.

## Figures and Tables

**Table 1:** Assignment of proton and carbon signals in the representative ^1^H-NMR spectra (CD_3_OD: D_2_O = 1:1).

No.	Metabolites	*δ*_H_	*δ*_C_
**1**	malic acid	2.67 (dd, *J* = 15.3, 3.0 Hz), 2.37 (dd, *J* = 15.3, 10.0 Hz), 4.28 (dd, *J* = 10.0, 3.0 Hz)	
**2**	citric acid	2.50 (d, *J* = 15.6 Hz), 2.69 (d, *J* = 15.6 Hz)	45.7, 75.1, 179.0, 182.0
**3**	formic acid	8.46 (s)	
**4**	succinic acid	2.41 (s)	
**5**	RC(OH)CH_3_-COOH	1.36 (s)	77.0, 180.9
**6**	lactic acid	1.34 (d, *J* = 6.8 Hz)	
**7**	fumaric acid	6.50 (s)	
**8**	sucrose	5.40 (d, *J* = 3.8 Hz), 4.17 (d, *J* = 8.7 Hz)	
**9**	*α*-glucose	5.21 (d, *J* = 3.8 Hz)	92.6
**10**	*β*-glucose	4.60 (d, *J* = 7.9 Hz)	96.3
**11**	fructose	4.09 (1H, d, *J* = 3.5 Hz)	77.6
**12**	alanine	1.49 (d, *J* = 7.3 Hz)	175.5, 50.5
**13**	threonine	1.32 (d, *J* = 6.6 Hz)	
**14**	mimosine	7.65 (overlapped, 2H), 6.54 (d, *J* = 6.7 Hz), 4.34 (dd, *J* = 14.2, 5.0 Hz), 4.23 (dd, *J* = 14.2, 6.7 Hz)	
**15**	quercetin	7.51 (d, *J* = 2.1 Hz), 7.47 (dd, *J* = 8.6, 2.1 Hz), 6.89 (d, *J* = 8.5 Hz), 6.31 (d, *J* = 1.8 Hz), 6.13 (d, *J* = 1.8 Hz)	
**16**	quercetin-3-*O*-*α*-rhamnoside	7.36 (d, *J* = 2.1 Hz), 7.32 (dd, *J* = 8.4, 2.1 Hz), 6.91 (1H, d, *J* = 8.4 Hz), 6.29 (d, *J* = 1.9 Hz), 6.13 (d, *J* = 1.9 Hz), 0.94 (d, *J* = 6.2)	
**17**	choline	3.22 (s)	
**18**	steroids	0.95 (d, *J* = 6.6 Hz), 0.92 (d, *J* = 6.7 Hz), 0.91 (d, *J* = 6.1 Hz), 0.95 (t, *J* = 7.5 Hz), 0.90 (t, *J* = 7.4 Hz)	134.2
**19**	3,4-dihydroxypyridine	7.69 (overlapped, 2H), 6.60 (d, *J* = 7.2 Hz)	
**20**	unidentified	2.09 (s)	
**21**	unidentified	2.72 (s)	

## Supplementary Materials

The following are available online, Figure S1: the growing *L. leucocephala* plants used in our research. Figure S2: the chemical structures of nineteen identified metabolites. Figure S3: the ^1^H- and ^13^C-NMR spectra of mimosine (**14**), quercetin (**15**), and quercetin-3-*O*-*α*-rhamnoside (**16**). The summary of fits (Figure S4) and the permutation plots (Figure S5) of PLS-DA model of JA, MeJA, JA-Ile, and CGM treatment. The relative contents of nineteen determined and two unidentified compounds in each treatment were shown in Table S1.

## Abbreviations

JA	jasmonic acid
MeJA	methyl jasmonate
CGM	2-[(6-ethyl-1-oxoindane-4-carbonyl) amino] acetic acid methyl ester
JA-Ile	jasmonoyl-l-isoleucine
NMR	nuclear magnetic resonance
PCA	principal component analysis
PLS-DA	partial least squares discriminant analysis
TSP	sodium salt of 3-trimethylsilylpropionic acid
