# Supplementary material for: Jasmonate-Elicited Stress Induces Metabolic Change in the Leaves of Leucaena leucocephala
Source: Molecules. 2018 Jan 24;23(2):188. doi: 10.3390/molecules23020188 (PMC6017012; doi:10.3390/molecules23020188)
Supplement: Supplementary file 1 [file molecules-23-00188-s001.zip › molecules-262884-supplementary/Supplementary Materials/Supplementary Materials of Figures S.pdf]

Figure S1. The growing plants of *L. leucocephala* in our research.

Figure S2. The chemical structures of nineteen identified metabolites. The notations of 1-19 were corresponding to different metabolites as listed in Table 1.

Figure S3. The ^{1}H and ^{13}C NMR spectra of mimosine (14), quercetin (15), and quercetin-3-*O*-α-rhamnoside (16).

Figure S4. The summary of fits of PLS-DA models of JA, MeJA, CGM and control, respectively, when two components were calculated.

Figure S5. The permutations plots of JA, MeJA, JA-Ile and CGM treatments were performed, respectively, in order to validate PLS-DA model, with 200 permutations and two components calculated.

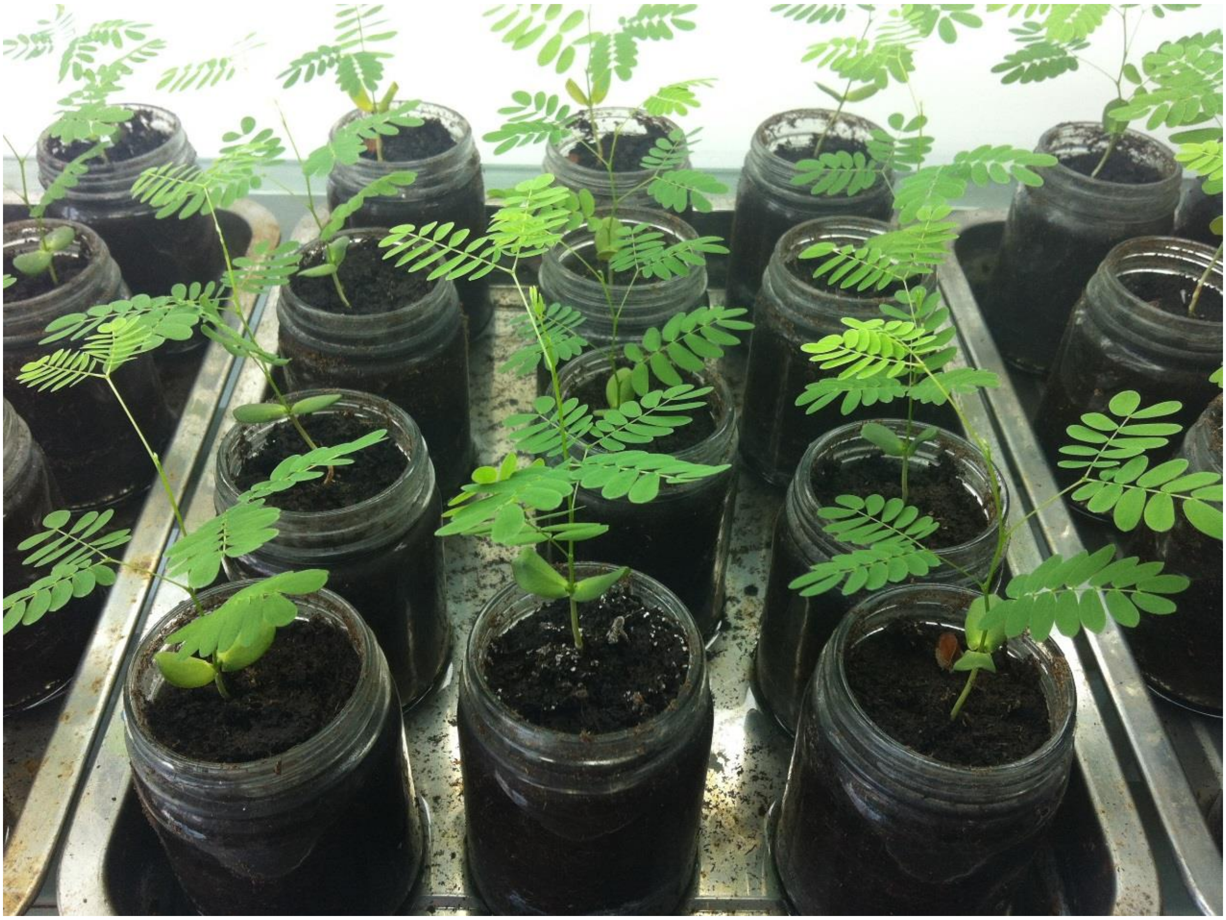

Figure S1

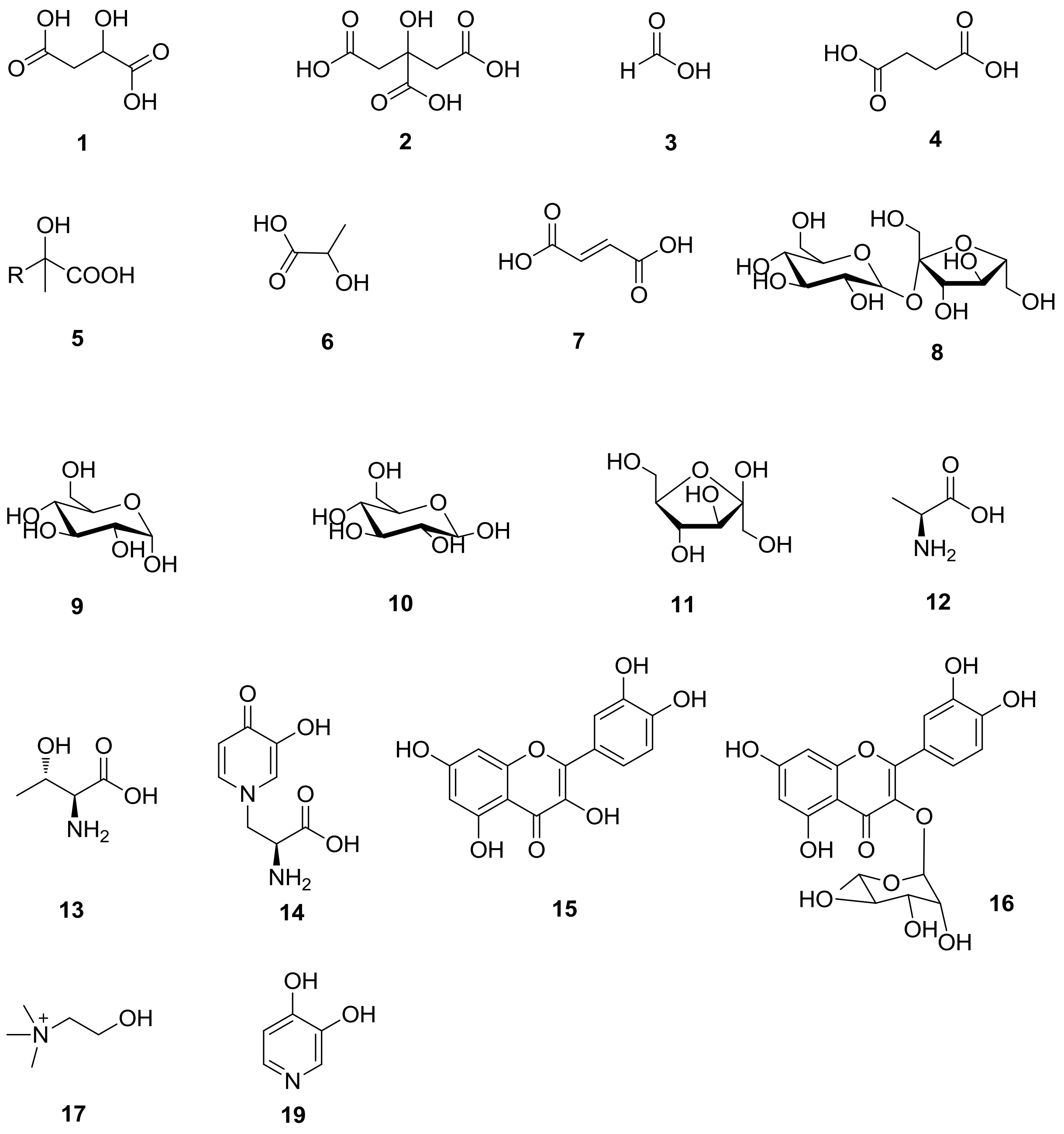

Figure S2

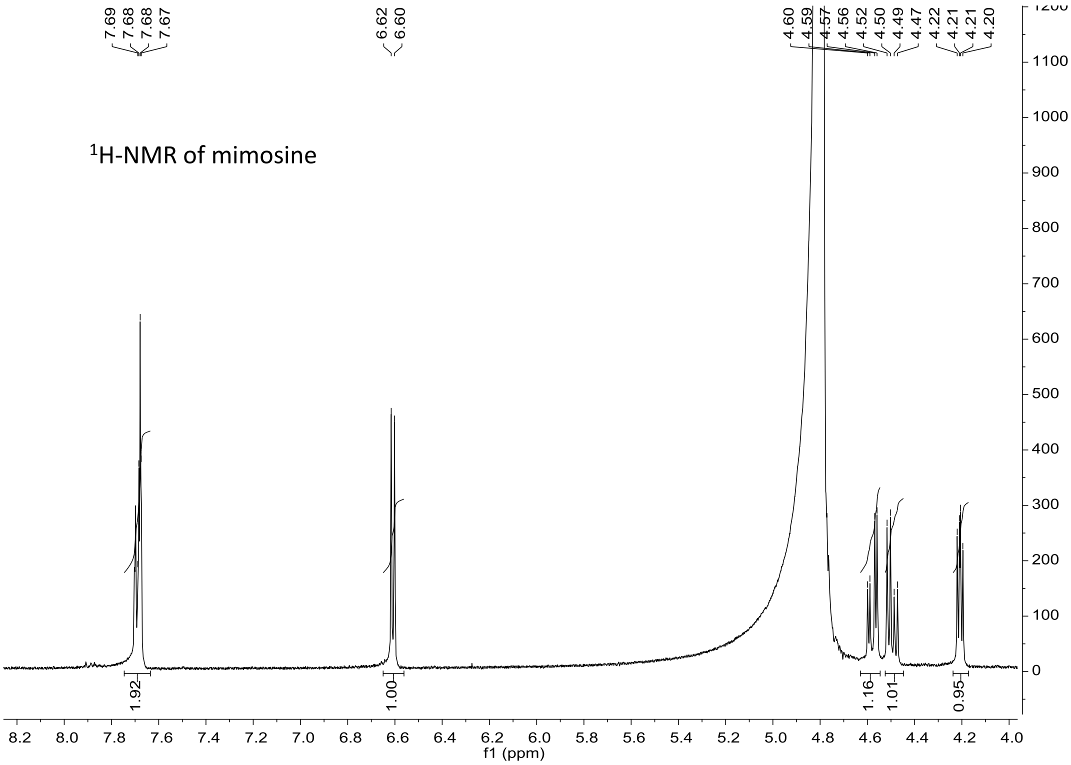

7.69
7.68
7.68
7.67
6.62
6.60
4.60
4.59
4.57
4.56
4.52
4.50
4.49
4.47
4.22
4.21
4.21
4.20
^{1}H-NMR of mimosine
1.92
1.00
1.16
1.01
0.95
f1 (ppm)

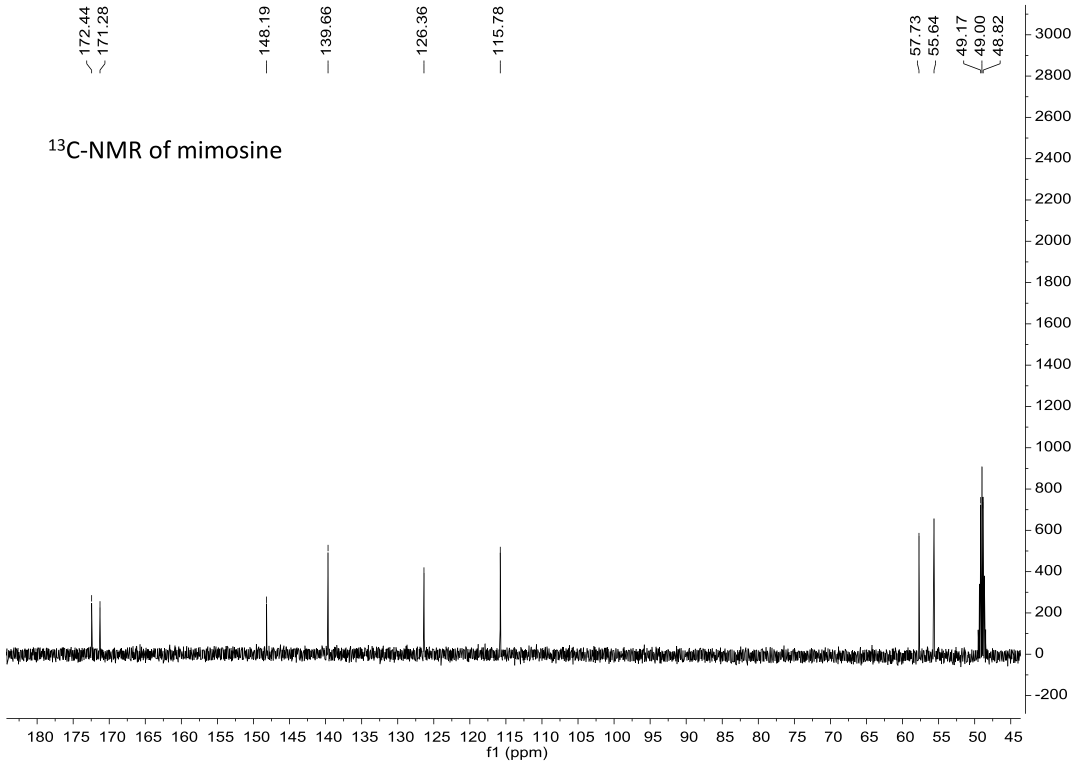

172.44
171.28
148.19
139.66
126.36
115.78
57.73
55.64
49.17
49.00
48.82
^{13}C-NMR of mimosine
f1 (ppm)

Figure S3

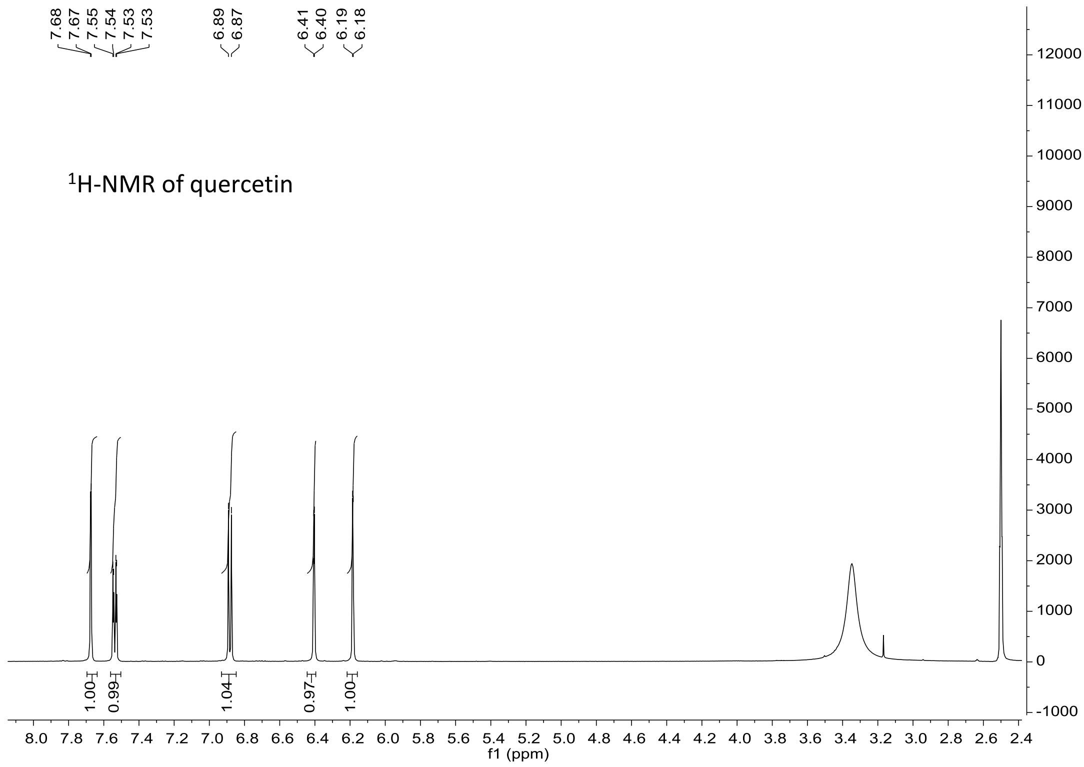
7.68
7.67
7.55
7.54
7.53
7.53
6.89
6.87
6.41
6.40
6.19
6.18
1H-NMR of quercetin
1.00
0.99
1.04
0.97
1.00
f1 (ppm)

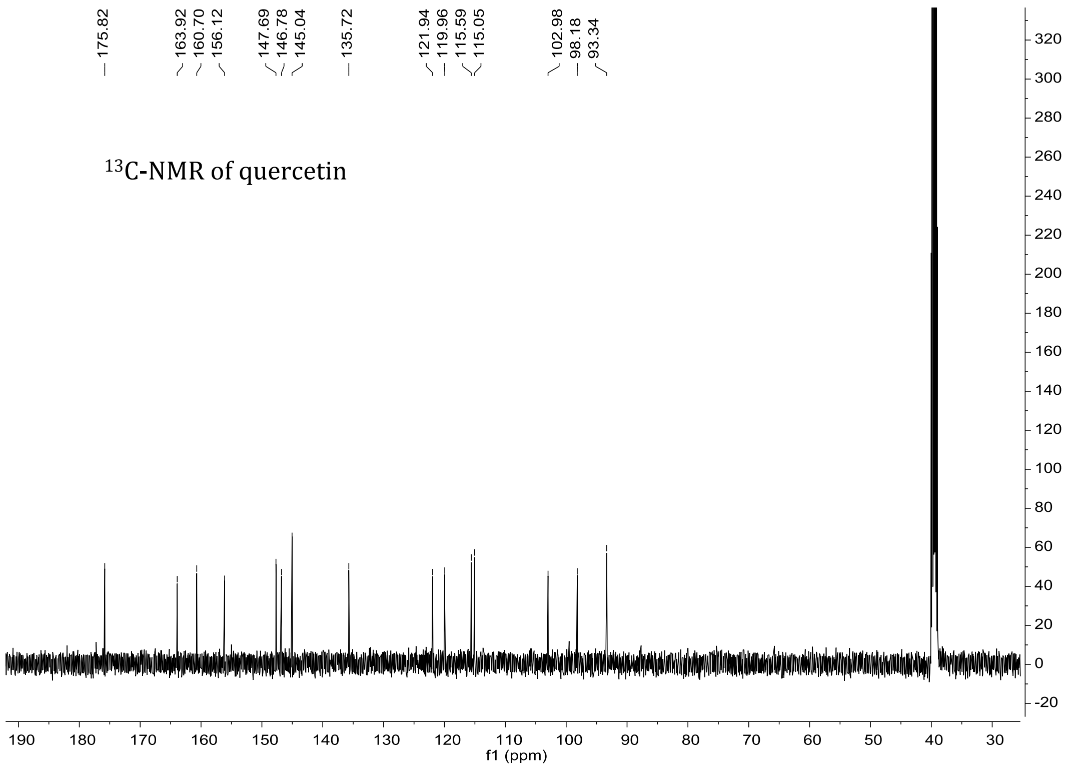
175.82
163.92
160.70
156.12
147.69
146.78
145.04
135.72
121.94
119.96
115.59
115.05
102.98
98.18
93.34
13C-NMR of quercetin
f1 (ppm)

Figure S3

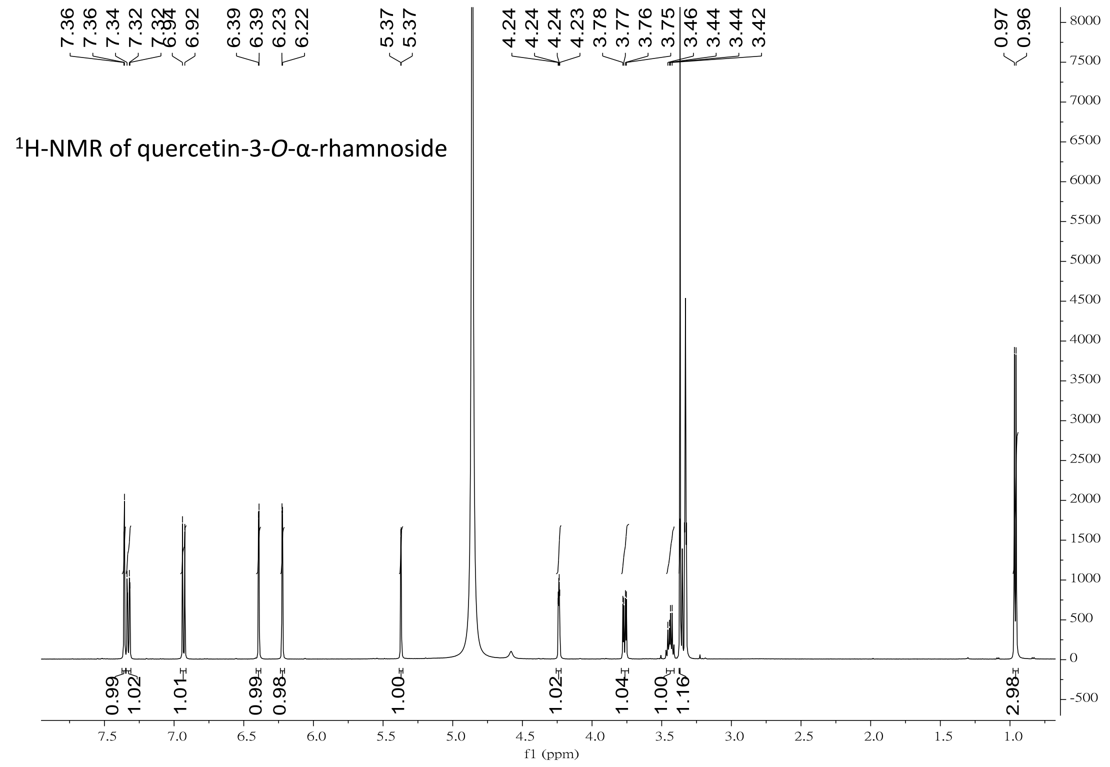

7.36 7.36 7.34 7.32 7.32 6.94 6.92 6.39 6.39 6.23 6.22 5.37 5.37 4.24 4.24 4.24 4.23 3.78 3.77 3.76 3.75 3.46 3.44 3.44 3.42 0.97 0.96
1H-NMR of quercetin-3-O-α-rhamnoside
0.99 1.02 1.01 0.99 0.98 1.00 1.02 1.04 1.00 1.16 2.98
f1 (ppm)

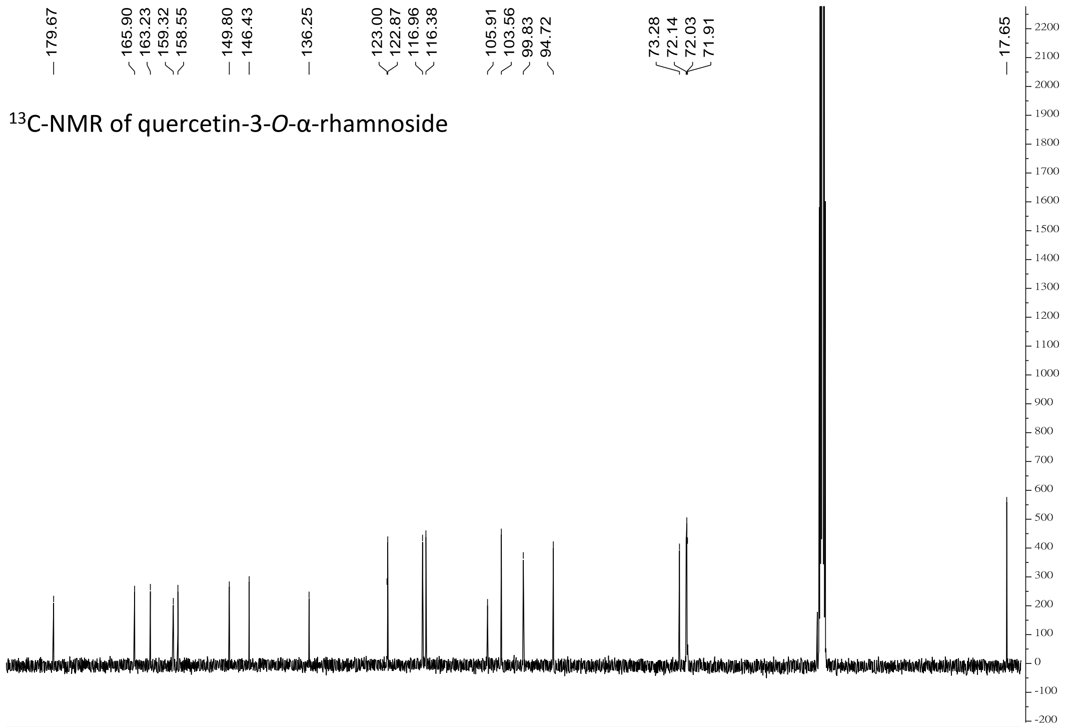

179.67 165.90 163.23 159.32 158.55 149.80 146.43 136.25 123.00 122.87 116.96 116.38 105.91 103.56 99.83 94.72 73.28 72.14 72.03 71.91 17.65
13C-NMR of quercetin-3-O-α-rhamnoside
f1 (ppm)

Figure S3

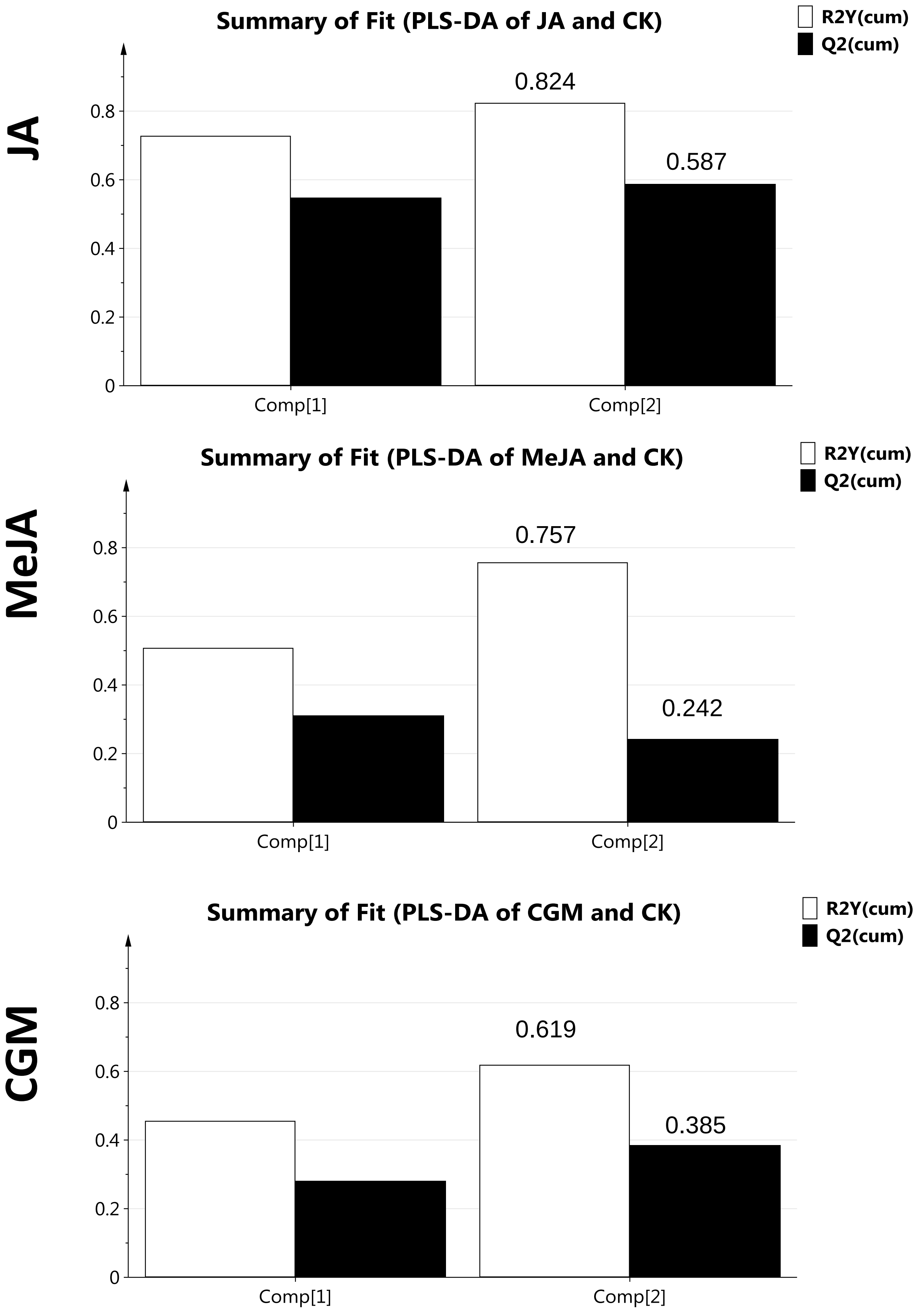

JA
Summary of Fit (PLS-DA of JA and CK)
R2Y(cum)
Q2(cum)
0.824
0.587
0
0.2
0.4
0.6
0.8
Comp[1]
Comp[2]
MeJA
Summary of Fit (PLS-DA of MeJA and CK)
R2Y(cum)
Q2(cum)
0.757
0.242
0
0.2
0.4
0.6
0.8
Comp[1]
Comp[2]
CGM
Summary of Fit (PLS-DA of CGM and CK)
R2Y(cum)
Q2(cum)
0.619
0.385
0
0.2
0.4
0.6
0.8
Comp[1]
Comp[2]

Figure S4

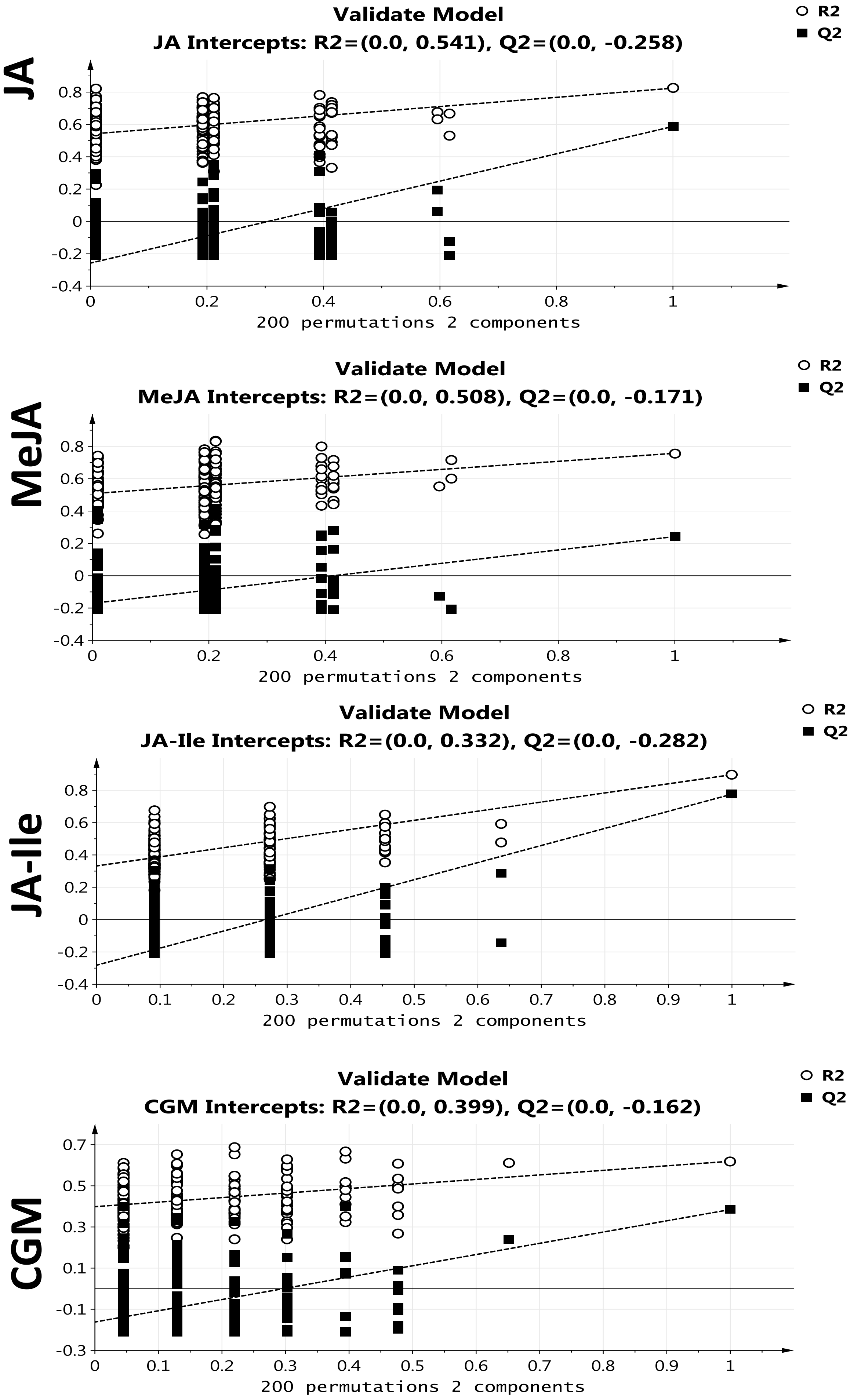

JA
Validate Model
JA Intercepts: R2=(0.0, 0.541), Q2=(0.0, -0.258)
R2
Q2
200 permutations 2 components
MeJA
Validate Model
MeJA Intercepts: R2=(0.0, 0.508), Q2=(0.0, -0.171)
R2
Q2
200 permutations 2 components
JA-Ile
Validate Model
JA-Ile Intercepts: R2=(0.0, 0.332), Q2=(0.0, -0.282)
R2
Q2
200 permutations 2 components
CGM
Validate Model
CGM Intercepts: R2=(0.0, 0.399), Q2=(0.0, -0.162)
R2
Q2
200 permutations 2 components

Figure S5
